# NeuroImages: IgG4-related disease with meningeal and parenchymal involvement on MRI and histopathology

**DOI:** 10.1007/s10072-025-08337-x

**Published:** 2025-07-10

**Authors:** Lina Li, Haibing Xiao

**Affiliations:** 1 Department of Neuromedicine Center, The University of Hong Kong-Shenzhen Hospital, Shenzhen, Guangdong China; 2 Shenzhen Clinical Research Center for Rare Diseases, Shenzhen, China

**Keywords:** IgG4-related disease, Central nervous system, Parenchymal involvement, Glucocorticoids

## Abstract

IgG4-related disease (IgG4-RD) rarely involves the CNS parenchyma. We report a 44-year-old man with left facial numbness and seizures. MRI showed right frontal-parietal dural thickening and parenchymal edema. Biopsy revealed IgG4 + plasma cells (> 50/HPF) with obliterative phlebitis, confirming IgG4-RD. This case highlights the need to consider IgG4-RD in meningo-parenchymal lesions mimicking tumors or infections. Glucocorticoids should be initiated promptly after histopathologic confirmation.

## Clinical presentation

A 44-year-old man presented with left facial numbness and focal seizures involving the left mouth corner. Neurologic examination revealed reduced pinprick sensation in the left trigeminal V2/V3 distribution.

## Imaging findings

**Fig. 1:**
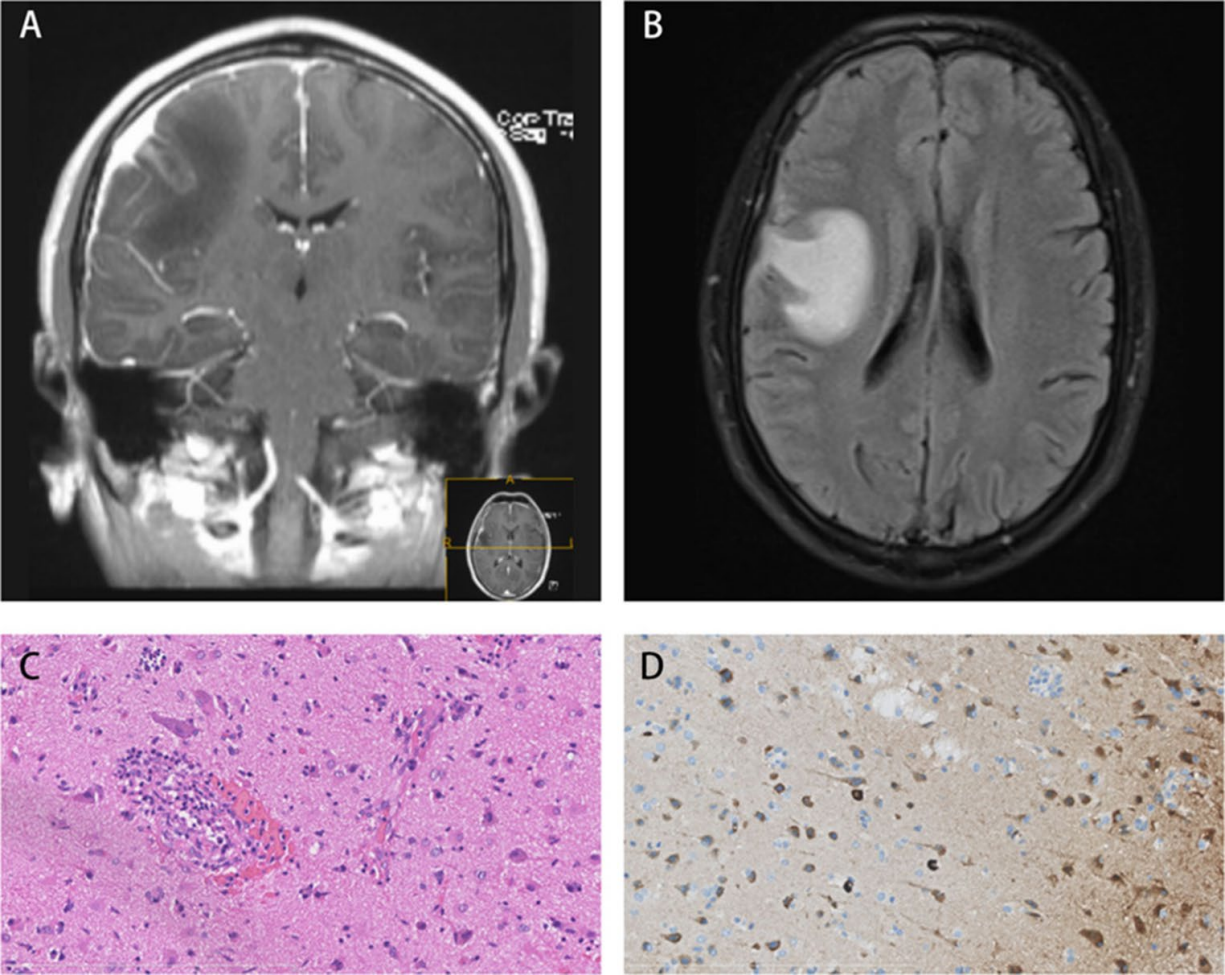
**A**: Axial brain MRI images demonstrate significant thickening and enhancement of the right frontal and parietal dura mater, accompanied by adjacent leptomeningeal enhancement and subtle ring enhancement within the brain parenchyma. **B**: FLAIR sequences display hyperintense patches in the adjacent right frontal and parietal lobes, consistent with edematous changes. **C**: Histopathological examination of the lesion reveals lymphoplasmacytic infiltrate and obliterative phlebitis within the parenchyma (Haematoxylin–Eosin stain, original magnification × 40). **D**: Immunohistochemical analysis highlights the presence of IgG4 + plasma cells in the parenchyma, a characteristic feature of IgG4-related disease (Immunohistochemistry for IgG4, original magnification × 40)

Thickened, enhancing dura mater in the right frontal-parietal region with adjacent leptomeningeal involvement. Parenchymal FLAIR hyperintensity and subtle ring enhancement, suggesting edema and inflammation (Fig. 1A, B).

## Pathology

Dense lymphoplasmacytic infiltrates with obliterative phlebitis (Fig. 1C, H&E).

IgG4 + plasma cells (> 50/HPF, IgG4/IgG ratio > 40%) (Fig. 1D, IHC), confirming IgG4-related disease (IgG4-RD).

## Teaching point

Immunoglobulin G4-related disease (IgG4-RD) of the central nervous system (CNS) primarily involves the dura mater and pituitary gland, but parenchymal infiltration (as in this case) is rare. This case underscores three key lessons:

Diagnostic Pitfall: IgG4-RD can mimic neoplasms (e.g., meningioma) or infections (e.g., tuberculosis) on MRI due to meningo-parenchymal lesions with enhancement.

Pathologic Hallmarks: Dense lymphoplasmacytic infiltrates (IgG4 + plasma cells ≥ 10/HPF and a IgG4 +/IgG + cell ratio > 40%), storiform fibrosis, and obliterative phlebitis are diagnostic [1].

Therapeutic Implication: Glucocorticoids and rituximab often lead to rapid symptom resolution, emphasizing the need for early biopsy [2].

## Data Availability

All data generated or analyzed during this study are included in this published article (and its supplementary information files). No additional external data repositories were used.
